# The Force Required to Detach a Rotating Particle from a Liquid–Fluid Interface

**DOI:** 10.1021/acs.langmuir.1c02085

**Published:** 2021-10-28

**Authors:** Abhinav Naga, Hans-Jürgen Butt, Doris Vollmer

**Affiliations:** Max Planck Institute for Polymer Research, Ackermannweg 10, 55128 Mainz, Germany

## Abstract

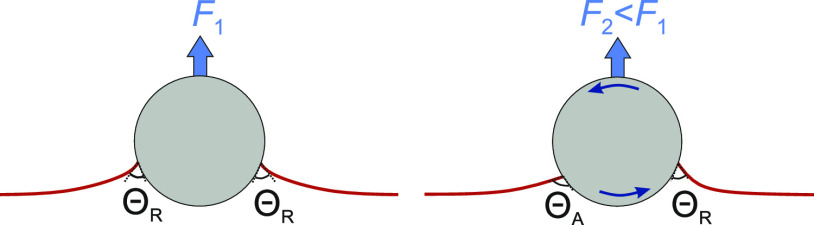

The force required to detach a particle from a liquid–fluid interface is a direct measure of the capillary adhesion between the particle and the interface. Analytical expressions for the detachment force are available but are limited to nonrotating particles. In this work, we derive analytical expressions for the force required to detach a rotating spherical particle from a liquid–fluid interface. Our theory predicts that the rotation reduces the detachment force when there is a finite contact angle hysteresis between the particle and the liquid. For example, the force required to detach a particle with an advancing contact angle of 120° and a receding contact angle of 80° (e.g., polydimethylsiloxane particle at a water–air interface) is expected to be 25% lower when the particle rotates while it is detached.

## Introduction

Capillary forces between particles and liquid–fluid interfaces have been studied experimentally, analytically, and numerically.^1−8^ Several materials and particle geometries have been investigated, including spheres, ellipsoids, and prisms.^3,9−14^ A direct measure of the adhesion between a particle and a liquid–fluid interface is the force required to detach the particle from the interface. For a spherical particle, this detachment force is given by^1,2^
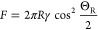
1where *R* is the radius of the particle, γ is the liquid–fluid interfacial tension, and Θ_R_ is the receding contact angle between the particle and the phase from which the particle is detached. Equation 1 is valid when the contact angle takes a single value, Θ_R_, throughout the three-phase contact line.

**Figure 1:**
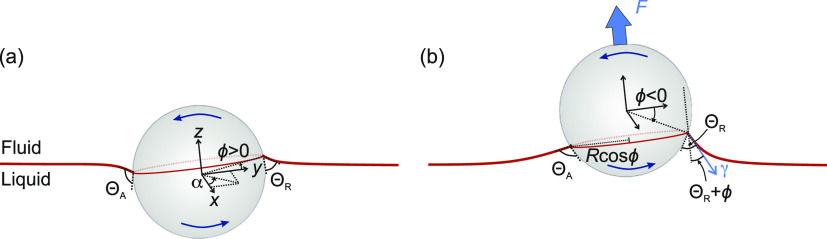
(a) Particle rotating against a liquid–fluid interface about the *x*-axis, which goes through its center. (b) Detaching a rotating particle by pulling it away from the liquid. The origin of the coordinate system is located at the center of the particle, and the *xy* plane is defined to be parallel to the three-phase contact line. α is the azimuthal angle in the *xy* plane. ϕ is the polar angle between the *y* axis and the three-phase contact line. ϕ is taken to be positive above the *xy* plane and negative below the *xy* plane.

However, in several scenarios, the contact angle does not take a single value along the entire contact line. For example, it has been shown that the contact angle around spherical and prismatic particles varies along their three-phase contact line^15−17^ and that the contact angle around a Janus particle varies around the contact line when the particle is oriented such that the boundary between the lyophobic and lyophilic hemispheres lies at a finite angle to an interface.^18^ Another example where the contact angle does not take a single value is when a particle rotates against a liquid–fluid interface, as shown in Figure 1a.^19^ There are several instances when rotation against an interface may become relevant: when a particle rolls down a wet or lubricated substrate,^20,21^ when a water drop removes contaminant particles from a substrate,^22^ when strong winds blow on soil or dust particles at the surface of a lake or river, and when a particle with an electric or magnetic dipole moment is placed at an interface in an electric or magnetic field, respectively.^23−28^ On one side of the rotational axis, the particle rolls out of the liquid, and therefore, the contact angle corresponds to the receding contact angle, whereas on the opposite side the contact angle corresponds to the advancing contact angle, Θ_A_.^19^ In general, Θ_A_ differs from Θ_R_ due to chemical and topographical inhomogeneities on the particle and/or adaptation of the particle to the liquid.^29,30^ Consequently, eq 1 does not hold for a rotating particle and should be modified.

In this work, we derive analytical expressions for the force required to detach a rotating spherical particle from a liquid–fluid interface. We compare predictions from four different models to test the sensitivity of the results to variations in the assumed contact line shape and contact angle variation around the contact line. Our theory predicts that it is easier to detach a particle from a liquid–fluid interface when the particle rotates against the interface during the detachment.

## Theory

In this section, we derive expressions for the force required to detach a particle from a liquid–fluid interface while it rotates about its center (Figure 1b). The second fluid can be a gas or a liquid that is immiscible in the first liquid. We assume that (1) the particle is small, such that gravity can be neglected, (2) the speed of rotation is small such that capillary forces dominate viscous forces, and (3) the speed of rotation is larger than the speed at which the center of mass moves relative to the interface during the detachment. Criterion (1) is valid as long as the particle’s radius, , where ρ is the density of the particle and *g* = 9.81 m s^–2^ is the gravitational acceleration (Supporting Information, S1). For example, the upper limit is R ≈ 1 mm for a glass particle (ρ ≈ 2500 kg m^–3^) at an air–water interface (γ = 72 mN m^–1^). Criterion (2) holds for small capillary numbers, η*v*/γ ≪ 1, where η is the dynamic viscosity of the more viscous fluid and *v* is the rotational speed at the surface of the particle (Supporting Information, S2). For example, for a particle at a water–air interface, criterion (2) is valid as long as *v* ≪ 100 m s^–1^. Criterion (3) implies that the contact angle on the side that rolls out of the liquid is equal to the receding contact angle and the contact angle on the side that rolls into the liquid is equal to the advancing contact angle throughout the detachment (Figure 1b).

The force required to overcome the capillary force (surface tension) and pull the particle away from the liquid, along the normal to the three-phase contact line is
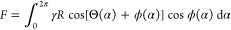
2Here, *R* is the particle’s radius, γ is the surface tension of the liquid, ϕ(α) is the polar angle between the *y* axis and the contact line about the center of the particle, α is the azimuthal angle in the *xy* plane, and Θ(α) is the contact angle between the liquid and the particle at an azimuthal angle α. The *xy* plane is chosen such that it goes through the center of the particle and is parallel to the plane containing the three-phase contact line. All the geometrical parameters are defined in Figure 1b.

**Figure 2:**
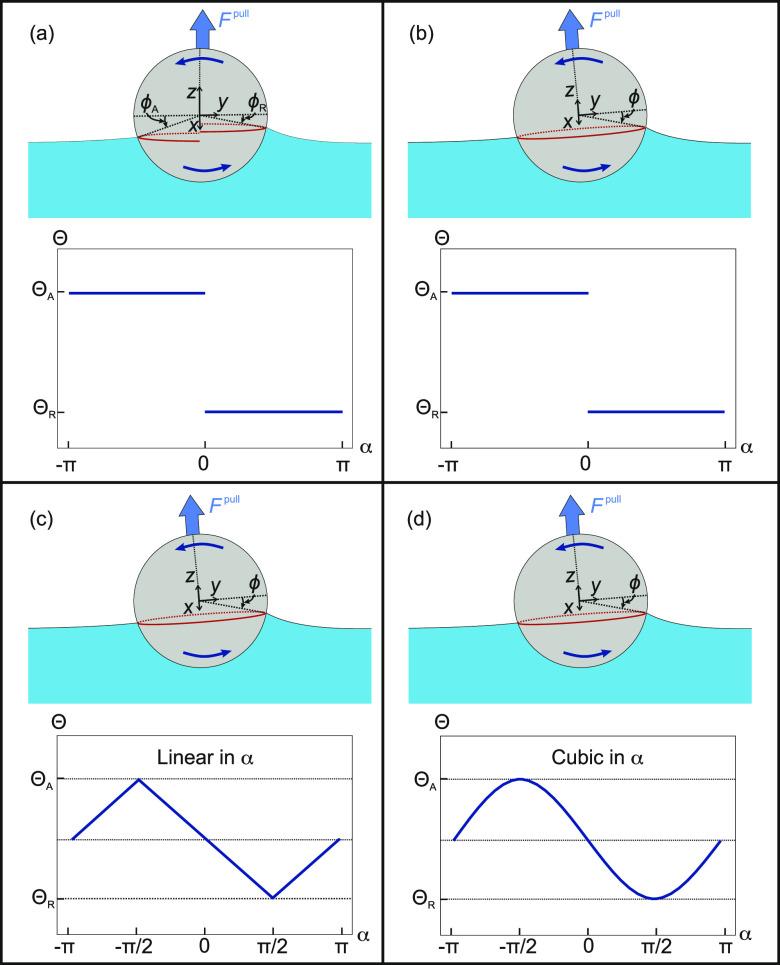
Schematics of the contact line shape and contact angle variation for the different models used. (a) Model 1 with a discontinuous contact line and a step contact angle variation. (b) Model 2 with a circular contact line and a step contact angle variation. (c) Model 3 with a circular contact line and a linear contact angle variation. (d) Model 4 with a circular contact line and a cubic contact angle variation. α is the azimuthal angle in the *xy* plane and *F*^pull^ is the force required to pull the particle out of the liquid.

In the absence of rotation, the contact angle has a single value around the contact line, and therefore, Θ(α) is independent of α. However, for a rotating particle, the contact angle varies along the contact line. Because the precise shape of the contact line and the variation of the contact angle around the contact line have never been imaged for a rotating particle, we consider four different models, each assuming a different contact line shape and contact angle variation. Model 1 assumes that the contact line is divided into two independent semicircles, each having a different contact angle (Figure 2a). Model 2 assumes a circular contact line and uses a Heaviside function to describe the contact angle variation (Figure 2b). Model 3 assumes a circular contact line and a linear variation of the contact angle (Figure 2c). Model 4 assumes a circular contact and a cubic variation of the contact angle (Figure 2d). Comparing the results from these four different models will allow us to test the influence of details of the contact line and contact angle variation on the detachment force.

We have derived expressions for the detachment force based on the assumptions of Model 1 in a previous work.^22^ However, Model 1 has limitations because it assumes a discontinuous contact line. In reality, the contact line should be smooth and continuous because any sharp discontinuity would imply an infinite Laplace pressure. Models 2, 3, and 4 are expected to be more realistic because they assume a continuous contact line. In the Supporting Information (Section S1), we derive expressions for the detachment forces based on the assumptions of Model 2. Below, we will focus on deriving analytical expressions based on the assumptions of Model 3 because it assumes a continuous contact angle variation, while also yielding analytical solutions. Model 4 cannot be solved analytically and therefore only numerical results will be presented.

According to Model 3, ϕ(α) is independent of α because the contact line is circular and the *xy* plane is defined to be parallel to the contact line. Θ(α) is given by
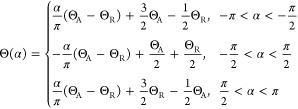
3

As the contact line and the contact angles are symmetric about the *y*-axis, we can evaluate eq 2 from α = – π/2 to α = π/2 and multiply the result by 2 to obtain the total force. Substituting eq 3 into eq 2 gives
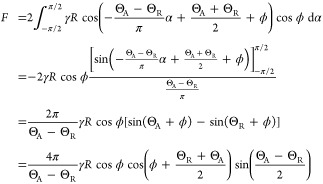
4

In reality, when a particle rotates at an initially horizontal interface, the three-phase contact line may be tilted by a finite angle τ to the horizontal. In the above derivation, we have defined the *xy* plane to be parallel to the contact line. Therefore, because the force given by eq 4 acts normal to the plane of the contact line, it may not point purely in the vertical direction but at an angle τ to it.

To completely detach the particle from the interface, the applied force has to exceed the maximum capillary force. As the particle is pulled away from the liquid, ϕ varies and a maximum force is obtained when d*F*/dϕ = 0 and d^2^*F*/dϕ^2^ < 0. Differentiating *F* with respect to ϕ gives
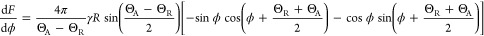
5d*F*/dϕ = 0 when the term in the square brackets is equal to zero. That is, when
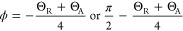
6

The first solution corresponds to a maximum (i.e., an upward force). Substituting ϕ = – (Θ_R_ + Θ_A_)/4 into eq 4 gives
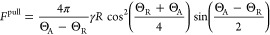
7

We call this force *F*^pull^ because it corresponds to the force required to detach the particle by pulling it away from the lower phase (liquid). The second stationary point [at ϕ = π/2 – (Θ_R_ + Θ_A_)/4] corresponds to the force required to detach the particle by pushing it into the liquid. The magnitude of this force is
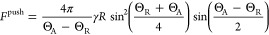
8

Note that eqs 7 and 8 are valid when Θ_A_ and Θ_R_ are expressed in radians. The results from all the models are summarized in Table 1.

**Table 1:** Maximum Capillary Forces on a Rotating Sphere Using Three Different Models for the Shape of the Contact Line and the Contact Angle Variation Around the Contact Line, as Described in Figure 2^a^

Model	*F*^push^	*F*^pull^
0	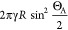	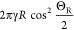
1	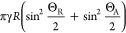	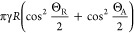
2	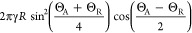	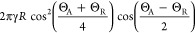
3	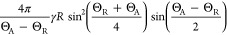	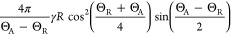

a Model 0 corresponds to a nonrotating particle. Model 4 is not included because it does not provide analytical expressions.

## Results and Discussion

**Figure 3:**
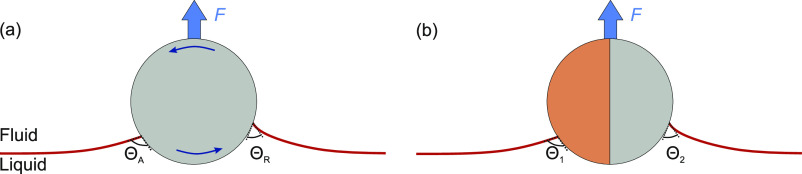
Scenarios having similar contact angle variation around the contact line. (a) Rotating homogeneous particle. (b) Nonrotating Janus particle oriented such that its lyophobic–lyophilic boundary lies perpendicular to the three-phase contact line.

The contact angle distribution around a rotating homogeneous particle is similar to that around a nonrotating Janus particle that is oriented such that its lyophobic–lyophilic boundary is perpendicular to the liquid–fluid interface (Figure 3). In both cases, the contact angle on one hemisphere differs from the contact angle on the opposite hemisphere. Therefore, the results in Table 1 (Models 1, 2, and 3) can also be applied to predict the force required to detach a (nonrotating) Janus particle when the lyophilic–lyophobic boundary lies perpendicular to the contact line. When applying the equations to a Janus particle, Θ_A_ and Θ_R_ have to be replaced by the contact angle that the liquid makes with the lyophobic and lyophilic sides, respectively.

In the following, we will focus on *F*^pull^ to analyze the consequences of rotation on the detachment force. The same conclusions also apply for *F*^push^.

### Comparison between Different Models

**Figure 4:**
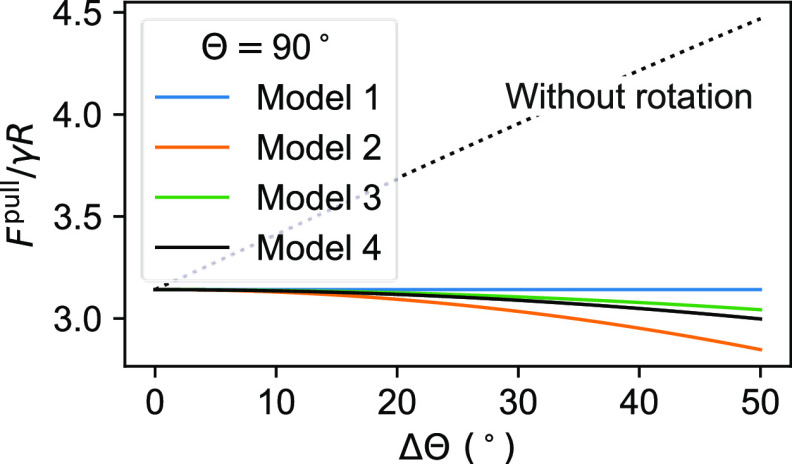
Comparison between the detachment force predicted by the models described in Figure 2 as a function of the contact angle hysteresis, ΔΘ = Θ_A_ – Θ_R_. The dotted black line shows the prediction by the model that ignores rotation (Model 0). All the lines are for Θ = (Θ_A_ + Θ_R_)/2 = 90°. Therefore, Θ_A_ = 90° + ΔΘ/2 and Θ_R_ = 90° – ΔΘ/2.

In Figure 4, we compare the predictions obtained for *F*^pull^ by the different models as a function of the contact angle hysteresis for an average contact angle of Θ = (Θ_R_ + Θ_A_)/2 = 90°. The prediction for Model 4 (circular contact line and cubic contact angle variation) was obtained by numerically integrating eq 2 with respect to α and maximising the result with respect to ϕ. The maximum force occurs when ϕ = – (Θ_R_ + Θ_A_)/4 for Models 2, 3, and 4. Because Model 1 assumes that the left and right hemispheres are completely independent, the maximum force predicted by Model 1 corresponds to a different value of ϕ on the right and left sides of the rotational axis. For Model 1, the maximum force occurs when ϕ = – Θ_R_/2 on the right and ϕ = – Θ_A_/2 on the left. The detachment forces predicted by Models 1, 2, 3, and 4 show little deviation from one another compared to their deviation from the detachment force of a nonrotating particle (Figure 4). This observation also applies to other values of Θ (Figures S1 and S2). In particular, Models 1, 2, and 4 deviate from Model 3 by less than 10% up to ΔΘ ≈ 55° and Θ = 100° (a range that includes most real materials). In most practical cases, particles are not completely uniform but have local impurities and defects. Hence, in reality, the shape of the contact line and the contact angle around the contact line may not precisely follow any of the geometries that we have assumed in Models 1, 2, 3, and 4 due to defects on the surface of the particle. However, because the detachment force predicted by the different models that assume different geometries only differ by around 10%, the derived expressions are expected to provide good estimates for the detachment forces even when the contact line is slightly distorted due to surface defects.

### Reduction in Detachment Force Due to Rotation

**Figure 5:**
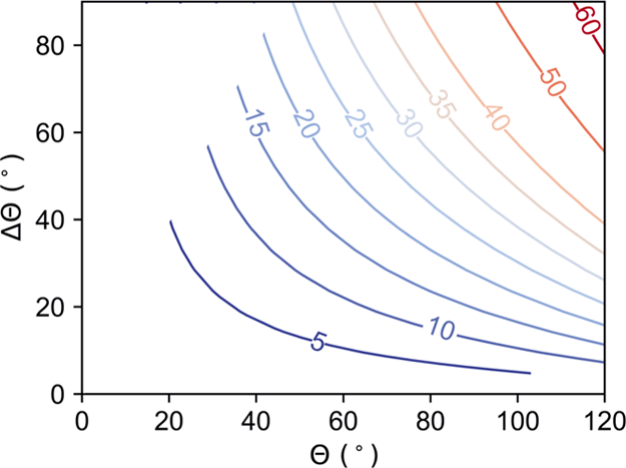
Percentage reduction in the detachment force when a particle rotates. Each line shows contours of constant percentage reduction. The numbers associated with each contour gives the reduction in force (in %), calculated using eq 9.

To quantify the influence of rotation on the detachment force, we compare the detachment force of a rotating particle with that of a nonrotating particle for a range of contact angles. The percentage reduction in the detachment force due to rotation is given by
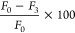
9where *F*_0_ corresponds to the detachment force of a nonrotating particle (Table 1, Model 0) and *F*_3_ corresponds to the detachment force of a rotating particle (Table 1, Model 3). Figure 5 shows contours of constant percentage reductions as a function of Θ and ΔΘ. We see the following: (i) for any fixed Θ, the percentage reduction increases with increasing ΔΘ, and (ii) for any fixed ΔΘ, the percentage reduction increases with increasing Θ. As an example, the force required to detach a polydimethylsiloxane (PDMS) particle from an air–water interface (Θ_A_ = 120° and Θ_R_ = 80°, γ = 72 mN m^–1^) is 25% lower when the particle rotates during the detachment.

Intuitively, the physical cause of the reduction in detachment force can be understood as follows. When a nonrotating particle is pulled out of a liquid, the effective contact angle is Θ_R_. In contrast, when the particle rotates during the detachment, the effective contact angle increases to (Θ_A_ + Θ_R_)/2. Therefore, rotation causes the particle to effectively appear more lyophobic relative to the fluid from which it is detached. As a result, it has a lower affinity for the fluid and hence can be detached more easily.

### Is It Beneficial to Use Rotation as a Means to Promote Detachment?

Because rotation causes a decrease in the detachment force, it may, at first, seem that inducing the rotation of particles could be a useful way to facilitate the detachment of particles from interfaces. However, this may not be economical from an energetic perspective because in order to rotate a particle against an interface, energy needs to be supplied to overcome resistive capillary torque (described in ref (19)). Capillary torque is due to the fact that the liquid–fluid interface is not axisymmetric about the center of a rotating particle. Therefore, the surface tension vector acts at different angles around the contact line, causing a net torque about the axis of rotation. The magnitude of the resistive capillary torque acting on a particle rotating at an interface is given by^19^
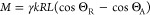
10where *k* ≈ 0.8 is a geometrical factor that depends on the shape of the contact line and the variation of the contact angle around the contact line and *L* is the diameter of the contact line. The energy that needs to be supplied to rotate the particle is 2π*M* per revolution. Therefore, it is only energetically economical to use rotation as a means to facilitate detachment if the energy saved by having a lower detachment force is greater than 2π*Mn*, where *n* is the number of revolutions before detachment occurs.

## Conclusions

Our theory predicts that the force required to detach a particle from a liquid–fluid interface is reduced when the particle rotates during the detachment. For example, the force required to detach a PDMS particle from an air–water interface is predicted to be 25% lower when the particle rotates while it is detached. The detachment force of a rotating particle depends on the advancing and receding contact angles between the liquid and the particle, the size of the particle, and the interfacial tension of the interface. Deviations due to the assumed shape of the contact line and the contact angle variation around the contact line affect the detachment force by only around 10%. Because 10% is relatively small for several practical scenarios, the forces predicted by the expressions derived in this paper will likely remain robust even if the contact line is slightly distorted due to inhomogeneities on the surface of the particle.

## Supporting Information Available

Comparison between the gravitational force and the capillary force acting on a particle, comparison between the viscous force and the capillary force acting on a rotating particle, derivation of expressions for the detachment force for Model 2, and comparison between the predictions obtained from the different models (PDF)
