# Supplementary material for: The Force Required to Detach a Rotating Particle from a Liquid–Fluid Interface
Source: Langmuir. 2021 Oct 28;37(44):13012–7. doi: 10.1021/acs.langmuir.1c02085 (PMC8582251; doi:10.1021/acs.langmuir.1c02085)
Supplement: Supplementary file 1 — la1c02085_si_001.pdf [file la1c02085_si_001.pdf]

Supporting Information for: The force required to detach a rotating particle from a liquid-fluid interface

Abhinav Naga, Hans-Jürgen Butt, and Doris Vollmer

Max Planck Institute for Polymer Research,

Ackermannweg 10, 55128 Mainz, Germany

October 27, 2021

S1 When is the gravitational force negligible compared to the capillary force?

The results presented in the manuscript are applicable for small particles, such that the gravitational force is negligible compared to the capillary force. For a particle at a liquid-fluid interface, the upper limit for the gravitational force is

$$F_g = \frac{4}{3}\pi R^3 \rho g \sim R^3 \rho g, \tag{S1}$$

where R is the radius of the particle, ρ is the density of the particle and g is the gravitational acceleration. Comparing F_g to a typical order of magnitude for the capillary force (γR) reveals that the capillary force dominates when

$$R < \sqrt{\frac{\gamma}{\rho g}}. \tag{S2}$$

For example, for a glass particle ($\rho \approx 2500$ kg m^3) at a water-air interface, the upper limit for R is approximately 1 mm.

Note that in general, the gravitational force, F_g will be even lower because buoyancy has to be subtracted, making the term of the right hand side of the inequality (Eq. S2) even larger and therefore increasing the upper limit for the particle size.

S2 When is the viscous force negligible compared to the capillary force?

An estimate for the viscous force can be obtained by considering a particle rotating inside a fluid about an axis going through its centre. The viscous torque (assuming a laminar flow) is given by [Eq. 1 in Lei *et al.* Appl. Phys. Lett. 89, 181908 (2006)]

$$M_{\mathrm{viscous}} = 8\pi\eta R^2 v, \tag{S3}$$

where η is the dynamic viscosity of the fluid, R is the radius of the particle and v is the speed of rotation at the surface of the particle. An effective viscous force (force corresponding to a couple with a torque M_{viscous}) can be obtained by dividing the viscous torque by the diameter of the particle. The effective viscous force is

$$F_{\mathrm{viscous}} = 4\pi\eta R v. \tag{S4}$$

Therefore, the viscous force scales as $\eta R v$.

In contrast, detachment force (capillary force) described in the manuscript scales as γR. Therefore, capillary forces dominate viscous forces when

$$\gamma R \gg \eta R v,$$

or equivalently, when

$$\frac{\eta v}{\gamma} \ll 1.$$

For example, for a particle at a water-air interface ($\gamma = 72$ mN m^{-1} and $\eta = 1$ mPa s), viscous effects can be neglected as long as $v \ll 100$ m s^{-1}.

S3 Derivation of detachment force for Model 2

In this section, we derive expressions for the detachment force assuming a circular contact line and a step variation in the contact angle [Fig. 2 (b)]. For this case, Eq. 2 becomes

$$\begin{aligned} F &= \int_0^{\pi} \gamma R \cos(\Theta_{\mathrm{R}} + \phi) \cos\phi \; d\alpha + \int_{-\pi}^{0} \gamma R \cos(\Theta_{\mathrm{A}} + \phi) \cos\phi \; d\alpha \\ &= \pi\gamma R \left[\cos(\Theta_{\mathrm{R}} + \phi) + \cos(\Theta_{\mathrm{A}} + \phi)\right] \cos\phi \\ &= 2\pi\gamma R \cos\left(\phi + \frac{\Theta_{\mathrm{A}} + \Theta_{\mathrm{R}}}{2}\right) \cos\left(\frac{\Theta_{\mathrm{R}} - \Theta_{\mathrm{A}}}{2}\right) \cos\phi. \end{aligned} \tag{S5}$$

To arrive at the last equation, the following trigonometric identity was used,

$$\cos(A + B) = 2\cos\left(\frac{A+B}{2}\right)\cos\left(\frac{A-B}{2}\right). \tag{S6}$$

To find the minimum and maximum values of F as ϕ is varied, we find the derivative of F with respect to ϕ,

$$\frac{dF}{d\phi} = -2\pi\gamma R \left[\cos\left(\phi + \frac{\Theta_{\mathrm{A}} + \Theta_{\mathrm{R}}}{2}\right)\sin\phi + \sin\left(\phi + \frac{\Theta_{\mathrm{A}} + \Theta_{\mathrm{R}}}{2}\right)\cos\phi\right]\cos\left(\frac{\Theta_{\mathrm{R}} - \Theta_{\mathrm{A}}}{2}\right). \tag{S7}$$

At the maximum/minimum points, $dF/d\phi = 0$, which is fulfiled when the terms in the square brakets in Eq. S7 is zero. That is when,

$$\begin{aligned}
\cos\left(\phi + \frac{\Theta_\mathrm{A} + \Theta_\mathrm{R}}{2}\right)\sin\phi &= -\sin\left(\phi + \frac{\Theta_\mathrm{A} + \Theta_\mathrm{R}}{2}\right)\cos\phi \\
\Rightarrow \tan\phi &= -\tan\left(\phi + \frac{\Theta_\mathrm{A} + \Theta_\mathrm{R}}{2}\right) \\
\Rightarrow \phi &= -\frac{\Theta_\mathrm{A} + \Theta_\mathrm{R}}{4} \text{ or } \frac{\pi}{2} - \frac{\Theta_\mathrm{A} + \Theta_\mathrm{R}}{4}.
\end{aligned} \tag{S8}$$

The first solution corresponds to the force required to detach the particle from the interface by pulling it away from the lower phase. Substituting $\phi = -(\Theta_\mathrm{A} + \Theta_\mathrm{R})/4$ in Eq. S5 gives

$$\begin{aligned}
F^\mathrm{pull} &= 2\pi\gamma R\cos\left(\frac{\Theta_\mathrm{A} + \Theta_\mathrm{R}}{4}\right)\cos\left(\frac{\Theta_\mathrm{R} - \Theta_\mathrm{A}}{2}\right)\cos\left(-\frac{\Theta_\mathrm{A} + \Theta_\mathrm{R}}{4}\right) \\
&= 2\pi\gamma R\cos^2\left(\frac{\Theta_\mathrm{A} + \Theta_\mathrm{R}}{4}\right)\cos\left(\frac{\Theta_\mathrm{A} - \Theta_\mathrm{R}}{2}\right).
\end{aligned} \tag{S9}$$

The second solution corresponds to the force required to detach the particle from the interface by pushing it into the lower phase. Substituting $\phi = \pi/2 - (\Theta_\mathrm{A} + \Theta_\mathrm{R})/4$ in Eq. S5 gives a force of magnitude

$$\begin{aligned}
F^\mathrm{push} &= -2\pi\gamma R\cos\left(\frac{\pi}{2} + \frac{\Theta_\mathrm{A} + \Theta_\mathrm{R}}{4}\right)\cos\left(\frac{\Theta_\mathrm{R} - \Theta_\mathrm{A}}{2}\right)\cos\left(\frac{\pi}{2} - \frac{\Theta_\mathrm{A} + \Theta_\mathrm{R}}{4}\right) \\
&= 2\pi\gamma R\sin^2\left(\frac{\Theta_\mathrm{A} + \Theta_\mathrm{R}}{4}\right)\cos\left(\frac{\Theta_\mathrm{A} - \Theta_\mathrm{R}}{2}\right).
\end{aligned} \tag{S10}$$

S4 Comparison between the different models

S4.1 Model 0 vs Models 1, 2, 3 and 4

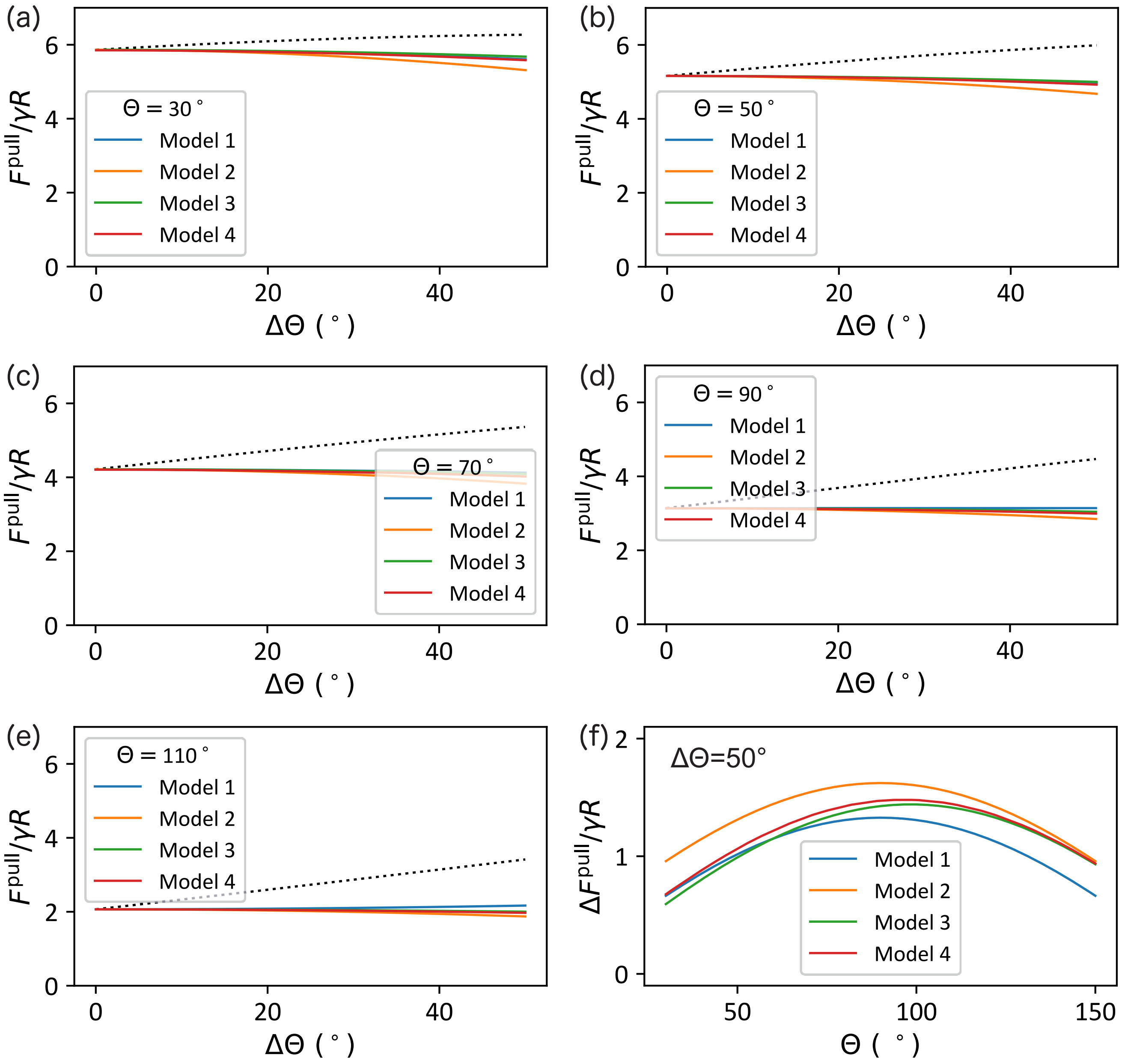

Figure S1: (a) - (e) Comparison of the detachment force, $F^{\rm pull}$, predicted by the different models as a function of contact angle hysteresis, $\Delta\Theta = \Theta_{\rm A} - \Theta_{\rm R}$ for different values of the average contact angle, $\Theta = (\Theta_{\rm A} + \Theta_{\rm R})/2$. The black dashed line is the force predicted if rotation is ignored. In (f), $\Delta F^{\rm pull} = F_0 - F_i$, where F_0 is the detachment force corresponding to a non-rotating particle and F_i is the detachment force for a rotating particle, as predicted by Models 1, 2, 3 or 4.

S4.2 Model 3 vs Models 1, 2 and 4

In this section, the detachment force (F^{pull}) predicted by Models 1, 2 and 4 are compared to the detachment force predicted by Model 3.

The percentage difference in the detachment force predicted by the models [Fig. S2] are calculated using

$$\frac{F_i - F_3}{(F_i + F_3)/2} \times 100, \tag{S11}$$

where F_i $(i = 1, 2, 4)$ is the detachment force predicted by Model 1, 2 and 4 respectively. F_3 is the detachment force predicted by Model 3.

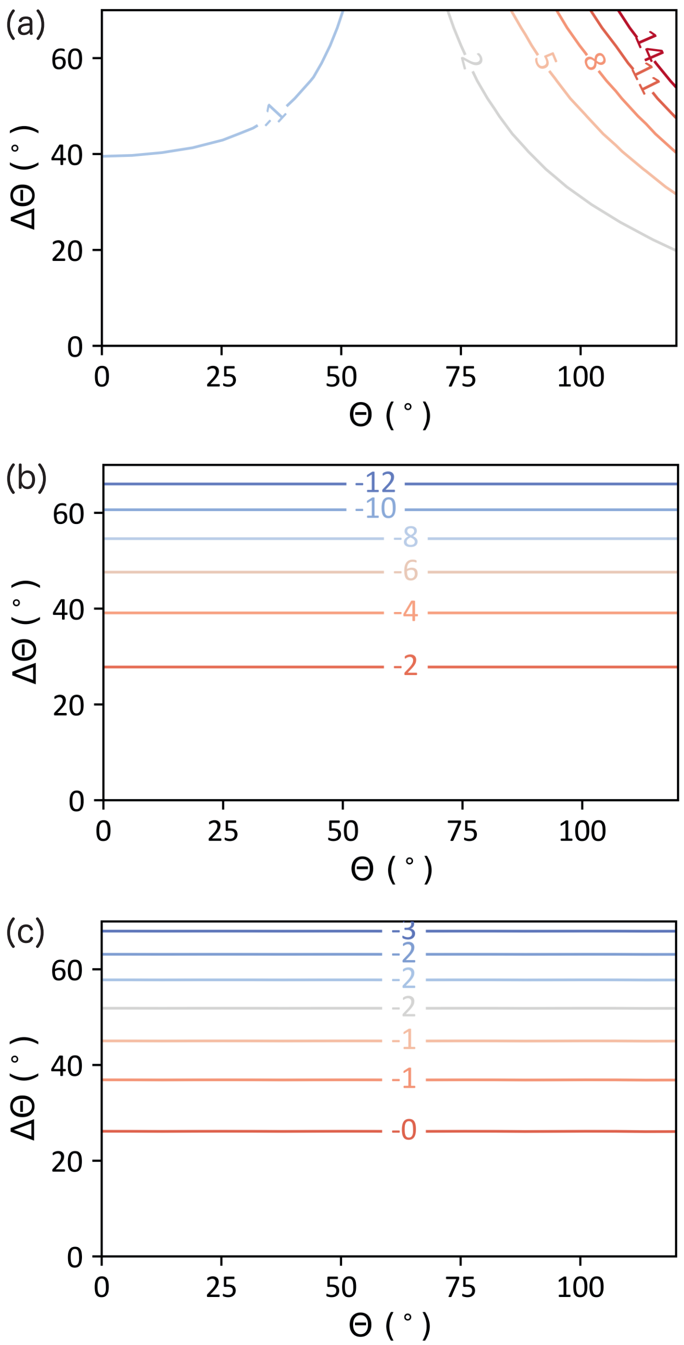

Figure S2: Comparison between the detachment force (F^{pull}) predicted by the different models. (a) Model 1 vs Model 3. (b) Model 2 vs Model 3. (c) Model 3 vs Model 4. The curves are contours of contact percentage difference, calculated using Eq. S11. The numbers on the contours are the percentage differences.
